# In vitro fertilization increases the odds of gestational diabetes: a nationwide register-based cohort study

**DOI:** 10.1007/s00592-022-01975-z

**Published:** 2022-10-22

**Authors:** Matias Vaajala, Rasmus Liukkonen, Ville Ponkilainen, Ville M. Mattila, Maiju Kekki, Ilari Kuitunen

**Affiliations:** 1 Faculty of Medicine and Life Sciences, University of Tampere, Tampere, Finland; 2 Department of Surgery, Central Finland Central Hospital Nova, Jyväskylä, Finland; 3 Department of Orthopaedics and Traumatology, Tampere University Hospital, Tampere, Finland; 4 Department of Obstetrics and Gynecology, Tampere University Hospital, Tampere, Finland; 5 Center for Child, Adolescent and Maternal Health Research, Faculty of Medicine and Health Technology, Tampere University, Tampere, Finland; 6 Department of Pediatrics, Mikkeli Central Hospital, Mikkeli, Finland; 7 Institute of Clinical Medicine and Department of Pediatrics, University of Eastern Finland, Kuopio, Finland

**Keywords:** In vitro fertilization, Gestational diabetes, Pregnancy

## Introduction

Assisted reproductive technology has been associated with an increased risk of gestational diabetes mellitus (GDM) [1, 2].However, when comparing intracytoplasmic sperm injection and in vitro fertilization (IVF), the risk has only been observed to be higher after IVF [1].

As the use of IVF is rapidly increasing [3], the adverse effects and complications caused by IVF should be better studied and acknowledged. Furthermore, current guidelines on GDM have no recommendations regarding IVF pregnancies [4]. As previous studies have been systematic reviews or have included relatively small populations, nationwide evidence with national guidelines and instructions on this topic is lacking. Hence, the aim of the present study was to evaluate, using nationwide registers, whether IVF is associated with a higher risk of developing GDM.

## Materials and methods

In this nationwide retrospective register-based cohort study, data from the National Medical Birth Register (MBR) were used to evaluate the risk of GDM caused by IVF. The MBR is maintained by the Finnish Institute for Health and Welfare. The study period was from 1 January 1998 to 31 December 2018.

The MBR contains data on pregnancies, delivery statistics, and the perinatal outcomes of all births with a birthweight ≥ 500 g or a gestational age ≥ 22^+0^ weeks, including fertility treatments such as IVF. GDM was diagnosed using the 75 g 2-h oral glucose tolerance test (OGTT). The MBR has high coverage and quality (the current coverage is nearly 100%).

**Fig. 1:**
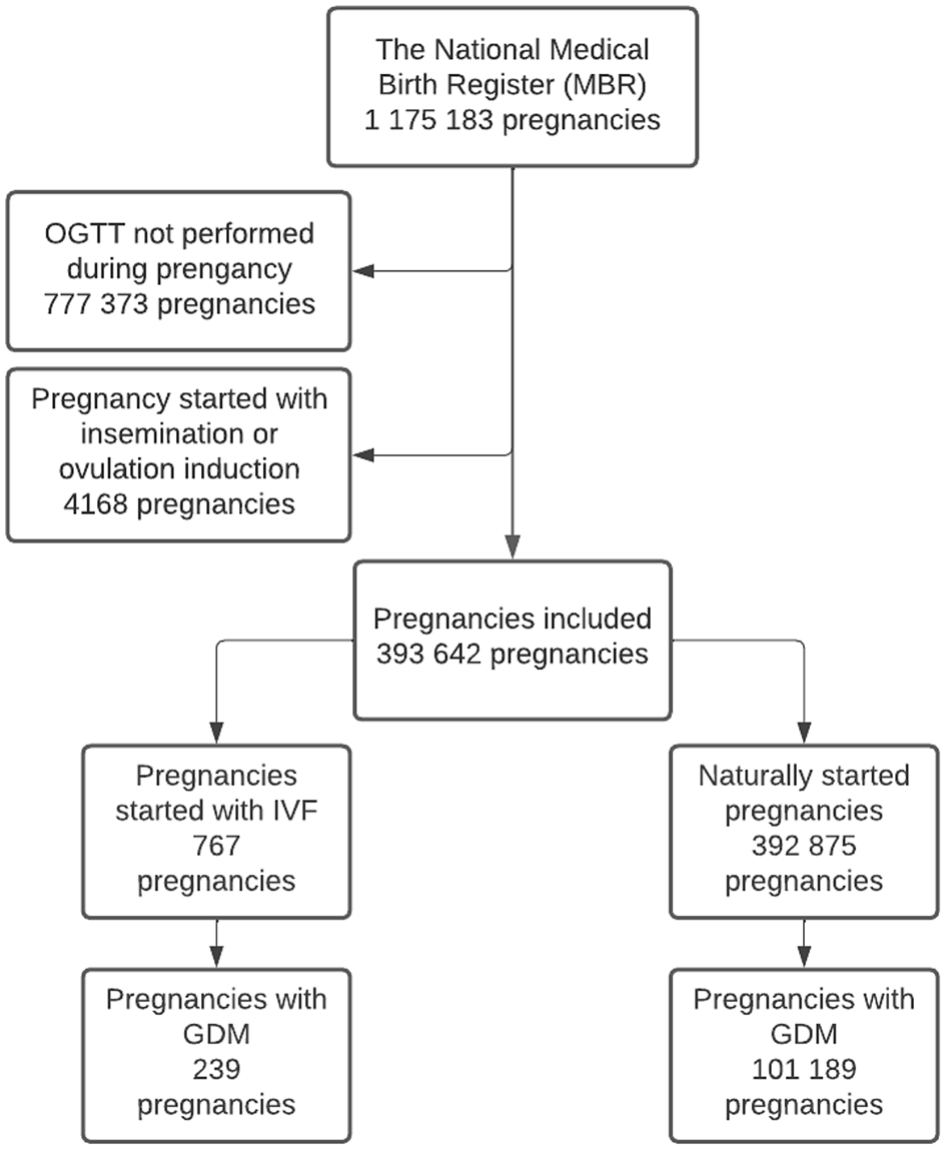
Flowchart of the study population. Pregnancies resulting from in vitro fertilization (IVF) were compared to naturally started pregnancies. Pregnancies in which 2-h oral glucose tolerance tests (OGTTs) were not performed or that were started with insemination or ovulation induction were excluded

We included all pregnancies in which the OGTT was administered in the MBR between 1998 and 2018. A total of 397 810 pregnancies were included in this study. Of these, 767 pregnancies that ended in delivery resulted from IVF and were included as the patient group. The reference group consisted of 392 875 naturally started pregnancies that ended in delivery. Pregnancies started with insemination or ovulation induction (*n* = 4168) were excluded from the reference group. The cohort selection is shown as a flowchart in Fig. 1.

The continuous variables were interpreted as means with standard deviations or as medians with interquartile ranges based on the distribution of the data. The categorical variables are presented as absolute numbers and percentages. Student’s *t*-test, the Mann–Whitney *U* test and Chi-squared tests were used for group comparisons. A logistic regression model was used to assess the primary outcome. The exposure variable was whether the pregnancy began naturally or with IVF, and the primary outcome was diagnosed GDM. Odds ratios (OR) and adjusted odds ratios (aOR) with 95% confidence intervals (CI) were compared between the groups. The model was adjusted for maternal age, continuous maternal body mass index (BMI), maternal smoking status, and the number of multiple pregnancies. Based on previous literature, all these are found to be risk factors for GDM [5].

## Results

A total of 239 GDM diagnoses were observed among the pregnancies started with IVF and 101 189 among the naturally started pregnancies (31.2% vs. 25.8%, *p* < 0.001). Maternal age was higher in the pregnancies started with IVF compared to the naturally started pregnancies (mean 33.9 years vs. 30.5 years, *p* < 0.001), while maternal BMI was lower in the group of pregnancies started with IVF (25.3 vs. 26.3, *p* < 0.001). A higher rate of mothers smoked in the naturally started pregnancies, while a higher rate of multiple pregnancies was observed among the pregnancies started with IVF (3.5% vs. 1.4%, *p* < 0.001) (Table 1). The adjusted odds for GDM were higher among the pregnancies started with IVF (aOR 1.26, CI 1.07–1.47) compared to the naturally started pregnancies.

**Table 1:** Background information on study groups

Total number	Pregnancy started with IVF		Pregnancy started naturally	
	767		392 875	
	*n*	%	*n*	%
Maternal age (mean; sd)	33.9 (4.7)		30.5 (5.3)	
BMI (mean; sd)	25.3 (4.5)		26.3 (5.4)	
BMI unknown	7	0.9	5105	1.3
Maternal smoking				
Smoking during pregnancy	28	3.6	56 240	14.3
Unknown	31	4.0	7925	2.0
Multiple pregnancy (> 1 fetuses)	27	3.5	5560	1.4
Gestational diabetes	239	31.2	101 189	25.8

## Discussion

The results of this nationwide study revealed that IVF is associated with higher odds of developing GDM. This association remained even after adjusting for possible confounding factors, such as maternal BMI, age, smoking and number of fetuses. Previously, IVF has been associated with a higher risk of GDM [1], and our study confirms this association by using nationwide high-quality registers.

Information gained from the present study is important as the incidences of IVF pregnancies, maternal obesity and GDM have continued to increase. It is important to recognize IVF as a risk factor for GDM, as it could mean the possibility of better prevention of GDM. Clinicians could also better inform patients about the possible GDM risk of IVF. As the current guidelines on GDM do not have recommendations regarding IVF [4], our results could be used to further evaluate whether the usage of IVF should be considered in the prevention of GDM and whether all IVF-started pregnancies should be screened for GDM by OGTT.

The strength of our study is the large nationwide register that includes data on OGTT testing and results for all pregnancies during the study period. The register data used in our study are routinely collected nationally in structured forms with consistent instructions, which ensures good coverage and reduces possible reporting and selection biases. The main limitation of our study is the missing clinical information on whether the patient had a history of either type I or type II diabetes, and this limitation should be acknowledged in the interpretation of the results. However, as our study sample was large and the usage of IVF is not related to a history of diabetes, we believe that this possible bias did not markedly affect our results. Another possible limitation for this study is that the screening methods for GDM changed after 2008 to comprehensive screening, meaning that the GDM testing rates increased notably towards the end of the study period.

In conclusion, the results of this study should be acknowledged by clinicians to recognize IVF as a risk factor for GDM, which is important in the prevention of GDM in the future.

## References

[1] Bosdou JK, Anagnostis P, Goulis DG (2020). Risk of gestational diabetes mellitus in women achieving singleton pregnancy spontaneously or after ART: a systematic review and meta-analysis. Hum Reprod Update.

[2] Pandey S, Shetty A, Hamilton M, Bhattacharya S, Maheshwari A (2012). Obstetric and perinatal outcomes in singleton pregnancies resulting from IVF/ICSI: a systematic review and meta-analysis. Hum Reprod Update.

[3] Kamphuis EI, Bhattacharya S, van der Veen F, Mol BWJ, Templeton A (2014). Are we overusing IVF?. BMJ.

[4] American Diabetes Association (2020) Management of diabetes in pregnancy: standards of medical care in diabetes-2020. Diabetes Care. Accessed 30 Aug 2022. https://diabetesjournals.org/care/article/43/Supplement_1/S183/30619/14-Management-of-Diabetes-in-Pregnancy-Standards

[5] Zhang Y, Xiao CM, Zhang Y (2021). Factors associated with gestational diabetes mellitus: a meta-analysis. J Diabetes Res.

